# OrthoDB in 2020: evolutionary and functional annotations of orthologs

**DOI:** 10.1093/nar/gkaa1009

**Published:** 2020-11-16

**Authors:** Evgeny M Zdobnov, Dmitry Kuznetsov, Fredrik Tegenfeldt, Mosè Manni, Matthew Berkeley, Evgenia V Kriventseva

**Affiliations:** Department of Genetic Medicine and Development, University of Geneva Medical School, rue Michel-Servet 1, 1211 Geneva, Switzerland, and Swiss Institute of Bioinformatics, rue Michel-Servet 1, 1211 Geneva, Switzerland; Department of Genetic Medicine and Development, University of Geneva Medical School, rue Michel-Servet 1, 1211 Geneva, Switzerland, and Swiss Institute of Bioinformatics, rue Michel-Servet 1, 1211 Geneva, Switzerland; Department of Genetic Medicine and Development, University of Geneva Medical School, rue Michel-Servet 1, 1211 Geneva, Switzerland, and Swiss Institute of Bioinformatics, rue Michel-Servet 1, 1211 Geneva, Switzerland; Department of Genetic Medicine and Development, University of Geneva Medical School, rue Michel-Servet 1, 1211 Geneva, Switzerland, and Swiss Institute of Bioinformatics, rue Michel-Servet 1, 1211 Geneva, Switzerland; Department of Genetic Medicine and Development, University of Geneva Medical School, rue Michel-Servet 1, 1211 Geneva, Switzerland, and Swiss Institute of Bioinformatics, rue Michel-Servet 1, 1211 Geneva, Switzerland; Department of Genetic Medicine and Development, University of Geneva Medical School, rue Michel-Servet 1, 1211 Geneva, Switzerland, and Swiss Institute of Bioinformatics, rue Michel-Servet 1, 1211 Geneva, Switzerland

## Abstract

OrthoDB provides evolutionary and functional annotations of orthologs, inferred for a vast number of available organisms. OrthoDB is leading in the coverage and genomic diversity sampling of Eukaryotes, Prokaryotes and Viruses, and the sampling of Bacteria is further set to increase three-fold. The user interface has been enhanced in response to the massive growth in data. OrthoDB provides three views on the data: (i) a list of orthologous groups related to a user query, which are now arranged to visualize their hierarchical relations, (ii) a detailed view of an orthologous group, now featuring a Sankey diagram to facilitate navigation between the levels of orthology, from more finely-resolved to more general groups of orthologs, as well as an arrangement of orthologs into an interactive organism taxonomy structure, and (iii) we added a gene-centric view, showing the gene functional annotations and the pair-wise orthologs in example species. The OrthoDB standalone software for delineation of orthologs, Orthologer, is freely available. Online BUSCO assessments and mapping to OrthoDB of user-uploaded data enable interactive exploration of related annotations and generation of comparative charts. OrthoDB strives to predict orthologs from the broadest coverage of species, as well as to extensively collate available functional annotations, and to compute evolutionary annotations such as evolutionary rate and phyletic profile. OrthoDB data can be assessed via SPARQL RDF, REST API, downloaded or browsed online from https://orthodb.org.

## INTRODUCTION

OrthoDB is a leading resource of evolutionary and functional annotations of orthologs (1). Orthology enables formulation of the most specific functional hypothesis for descendant genes (2,3), and therefore it is the preferred way for tentative characterization of genes in newly sequenced organisms. Orthology is also the cornerstone for comparative evolutionary studies. Initially coined for a pair of species, the term orthologs referred to genes that derive from a single gene in the last common ancestor (LCA) of the species (4). Generalization of the term to more than two species, however, required the LCA to be specified and led to the concepts of groups of orthologs or orthogroups (5) and levels of orthology (6,7). Although orthologs are clearly defined evolutionarily as descendant genes from a single gene of the LCA of the considered organisms, practical delineation of orthologs in extant species can be challenging as there are limitations to the accuracy with which gene and species genealogies can be reconstructed. Large-scale delineation of gene orthology is an even more demanding and challenging task, as there is a trade-off between accuracy and scalability of methods for making evolutionary inferences. The demand for automated methods for ortholog prediction is growing, and the challenges are exemplified by the numerous approaches proposed (6,8–16). Orthologer is the software behind the delineation of orthologs in OrthoDB that is freely available for standalone computations.

The concept of orthology is inherently hierarchical, as each phylogenetic clade or subclade of species has a distinct common ancestor, leading to more finely-resolved orthologs for more closely related species. OrthoDB has explicitly emphasized this since its inception (7), defining levels of orthology at each major radiation of the species taxonomy. Overcoming the original pair-wise thinking of orthology however is tangled and many find navigation to the most relevant level of orthology confusing. This prompted us to enhance the OrthoDB web interface to improve usability and simplify access to the ever-growing database.

Evolutionary and functional annotations of orthologs, predicted across thousands of genomes from all forms of life including viruses, remains a key feature of OrthoDB. *Functional annotations* of genes were extensively collated from major resources including Uniprot (17) and NCBI (18) gene records, as well as InterPro (19) Gene Ontology (GO) (20). Notably, we collated and made searchable references such as to KEGG (21) pathways and to Online Mendelian Inheritance In Man (OMIM^®^) (22) linking to human diseases. All data were processed to assemble consolidated and non-redundant per-gene annotation records, presented as short one-line descriptions. Consequent aggregations to the orthogroup-level from the corresponding gene-level annotations allowed us to compile: (a) one-line orthologous group descriptors to concisely but precisely outline functional knowledge in a human-readable language as described in an earlier publication (23) whenever possible assign functional categories of COG (24), GO, KEGG pathways and the enzyme EC number. Such high-level functional descriptors are informative for comparative studies and metagenomics. Functional annotation of genes is complicated and may contain errors. Discordant annotations should be considered with caution, and OrthoDB makes such errors in the underlying data apparent.

*Evolutionary annotations* were computed for each orthologous group from the available genomics data and sequence alignment metrics. As detailed earlier in (23) metrics include: ‘phyletic profile’ that reflects gene universality (proportion of species with orthologs) and duplicability (proportion of multi-copy versus single-copy orthologs), ‘evolutionary rate’ that reflects the relative constraints on protein sequence conservation or divergence, and ‘sibling groups’ that reflects the sequence uniqueness of the orthologs. The universality of a gene family hints at a functional load that is widely necessary and basal, while lineage-restricted genes may underlie lineage-specific adaptations. Duplicability is also indicative of the type of molecular functions, e.g. members of a signal-transduction pathway or a protein complex may evolve under the single-copy control (25). ‘Evolutionary rate’ is a relative measure of sequence divergence, where faster-than-average genome evolution may indicate positive selection. The ‘sibling groups’ allow navigation to gene families possibly having similar molecular functions. These annotations, which provide an evolutionary perspective, remain unique to OrthoDB.

The OrthoDB resource is public, including both data and data processing software. The optional registration allows the authenticated users to upload their own proteomic data, for example from freshly sequenced genomes, for performing online BUSCO (26) analysis and for mapping to the current OrthoDB data. This enables the user to map existing functional annotations to the new genes, as well as to generate user-tailored comparative charts depicting the total gene count, the fraction of common genes, the fraction of the most conserved single-copy genes, etc.

## COVERAGE OF ORGANISMS

In 2020, OrthoDB continues to provide the worldwide leading coverage of organisms. It notably includes Viruses and is set to further increase the sampling of Bacteria three-fold in release 11. The actual statistics of the analyzed organisms, their taxonomic relations, and the genome assembly accession numbers are shown and searchable under the ‘Advanced’ section of the user interface. The orthology levels, referring to the last common ancestors from which extant orthologs evolved, are defined according to the NCBI Taxonomy (27). Protein-coding gene translations are retrieved mostly from RefSeq and NCBI complete genomes (28). The number of sequenced genomes continues to grow exponentially, and it is important to uniformly cover the emerging diversity of sequences. This will not only facilitate studies of these organisms, but will also empower resolution of complex ortholog genealogies, with multiple gene duplications and losses. Sampling of species diversity was shown to be a major factor affecting the accuracy of inferred gene orthology, besides the quality of the underlying genomes and their annotations (29). OrthoDB collects available complete genomes, identifies well-sampled taxonomic units having over 96% pair-wise genomic identity using MASH (30), and then selects the most annotated and complete representative genomes for each taxonomic unit.

## WEB INTERFACE

The OrthoDB user interface has been enhanced to respond to the massive growth in available data There are now three views on the data: (i) a sorted list of ortholog groups resulting from free-text search against all identifiers and textual description records by user query, (ii) a detailed view of an ortholog group and (iii) a gene-centric view.

**Figure 1.**
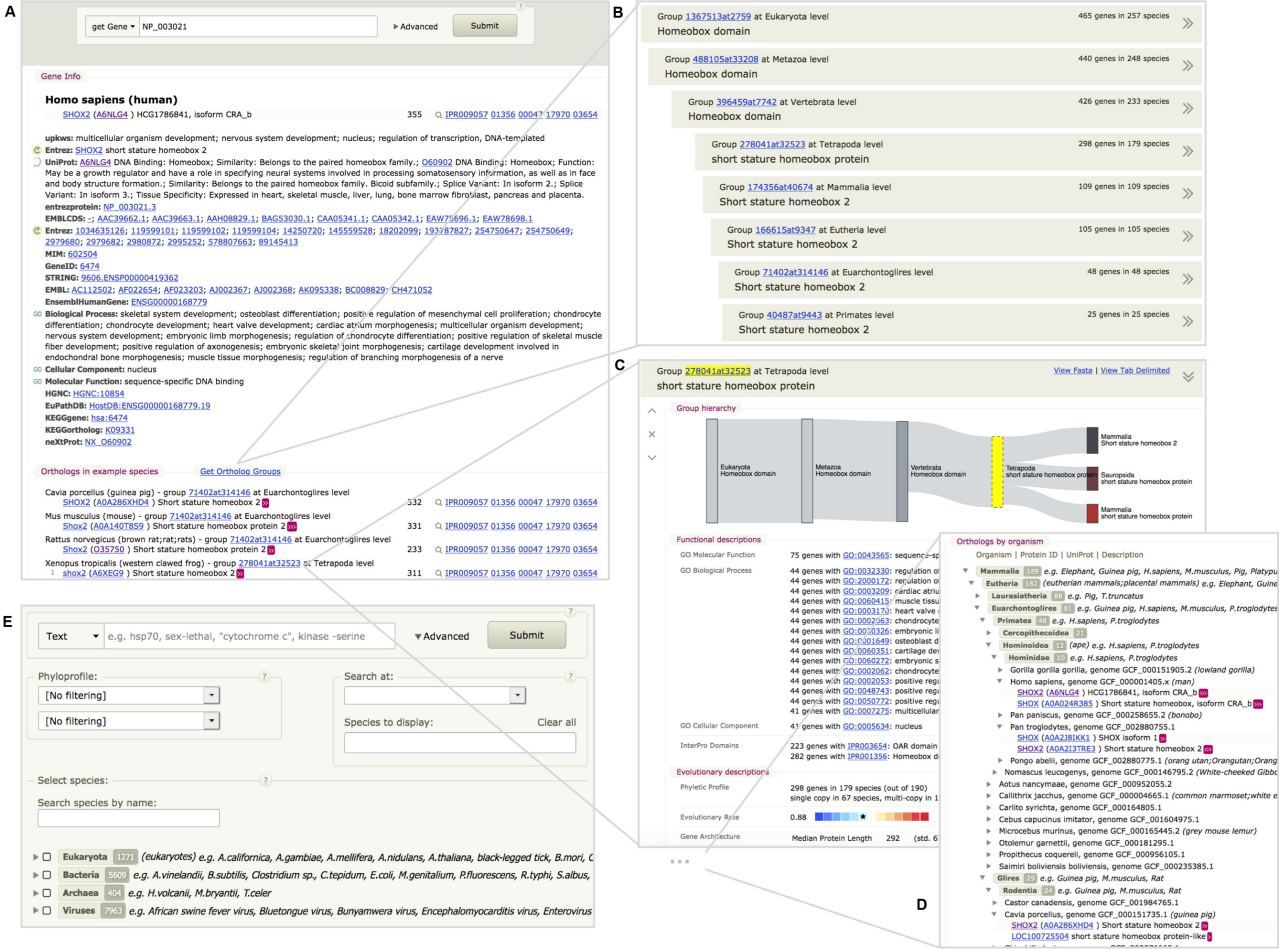
Elements of OrthoDB user interface include: (**A**) a gene-centric view, providing available gene annotations and a list of pair-wise orthologs in example species; (**B**) a hierarchically arranged list, sorted by relevance of ortholog groups matching the user query; (**C**) detailed view of an ortholog group, facilitating the navigation between the levels of orthology with a Sankey flow diagram and (**D**) arranging the list of orthologs into an interactive organism taxonomy structure to focus only on relevant data; (**E**) the ‘Advanced’ section of the web interface enables user-tailored selection of organisms to focus on, specifying explicitly relevant levels of orthology, or phyloprofile filters.

Since the last OrthoDB publication:

- the retrieved list of ortholog groups is now arranged to visualize their hierarchical relations. Previously, when a user searched by a keyword, an identifier or a complex query, OrthoDB returned a list of ortholog groups sorted only by textual relevance (i.e. a count of the number of query matches per group). The list remained unsorted if a single gene identifier pin-pointed one gene in one organism, e.g. if a Uniprot id or NCBI gid was queried. Since even simple queries by a gene identifier return several ortholog groups corresponding to different levels of orthology, for example across Eukaryotes, vertebrates, mammals etc., such an unsorted list can be confusing. In the revised interface we opted for double-sorting, firstly by textual relevance, then by the taxonomic level, from the more general to the more specific. The hierarchy is visualized by a ladder-like shifted list of ortholog group banners. This results in a more biologically intuitive output when OrthoDB is queried by a particular gene identifier, for example via a URL coming from NCBI linkouts (Figure 1B).
- we added a Sankey diagram to the detailed view of an ortholog group. This flow diagram shows the ortholog group within the hierarchy of the evolutionarily related groups, where the flow width is proportional to the number of genes in each group. This naturally facilitates visual navigation between the levels of orthology (Figure 1C). We also arranged the member genes by organism taxonomy (Figure 1D), which can be interactively expanded or collapsed. There are now many ortholog groups containing hundreds to thousands of genes, making them cumbersome to browse. Since many users are only interested in a small subset of available species, or specific taxonomic clades, this rearrangement is critical for OrthoDB usability, in that it hides most of the unnecessary data, while being intuitive and transparent for orthologs in other organisms. The default choice of expanded species can be customized by users in the ‘Advanced’ settings panel (Figure 1E). Each gene is further annotated with a wealth of collated functional descriptions and cross-references to other public resources that are hidden by default and can be viewed by clicking on ‘>>’ (where the size of the chevrons reflects the amount of available annotations).
- we added a gene-centric view, providing a list of pair-wise orthologs in example species in addition to the available annotations. Although since its inception OrthoDB explicitly emphasized groups of orthologs and their hierarchical nature (7), the notion of pair-wise orthology (i.e. between two species) is commonly used, and we hope this gene-centric view will facilitate bridging these two concepts. The pair-wise orthology in example species is arranged by the phylogenetic distance to the organism in which the gene matching the query was found. These are consequently linked to the corresponding groups of orthologs at the closest level of orthology to enable navigation to orthologs in the other organisms.

## ORTHOLOGER SOFTWARE

Orthologer, the software used to delineate orthologs in OrthoDB, is freely available for standalone computations (see more from https://www.orthodb.org/orthodb_userguide.html#standalone-orthologer-software). It is based on pair-wise assessments of protein sequence homology between complete genomes using MMseqs2 (31) and subsequent clustering, as previously described in (23). The latest adjustment to Orthologer includes the addition of an optional mode to perform hierarchical computation of orthology guided by a user-provided organism taxonomy.

## DATA AVAILABILITY

As for previous versions of OrthoDB we provide data files for bulk download, one file per level of orthology; as well as the underlying amino acid gene translations. To retrieve substantial subsets of data from OrthoDB or to access it programmatically we provide a REST API, documented at https://www.orthodb.org/orthodb_userguide.html#api, that returns data in *JSON*, *FASTA* or *TAB* formats. All data are distributed under the Creative Commons Attribution 3.0 License from http://www.orthodb.org/.

The RDF SPARQL interface was introduced in 2016 and it is gaining momentum. Adopting URIs of UniProt proteins and Ensembl genes, to be compatible with both UniProt and Ensembl SPARQL endpoints, it provides the possibility for very elaborate queries and a number of clickable links to Ensembl Genomes, NCBI, Interpro and GO resources. Users can also navigate to OrthoDB records by following links from FlyBase ‘Orthologs’ section, UniProt ‘Phylogenomic databases’ section, or NCBI ‘Additional links/ Gene LinkOut’ section.

## CONCLUSIONS AND PERSPECTIVES

The growing number of sequenced genomes increases the power of comparative analyses, but it also presents challenges regarding scalability of methods and data presentation to end users. OrthoDB strives to uniformly sample the available genomic space and to refine the accuracy of ortholog delineations.
